# Low-Coherence Interferometric Fiber-Optic Sensors with Potential Applications as Biosensors

**DOI:** 10.3390/s17020261

**Published:** 2017-01-28

**Authors:** Marzena Hirsch, Daria Majchrowicz, Paweł Wierzba, Matthieu Weber, Mikhael Bechelany, Małgorzata Jędrzejewska-Szczerska

**Affiliations:** 1 Department of Metrology and Optoelectronics, Faculty of Electronics, Telecommunications and Informatics, Gdańsk University of Technology, Narutowicza Street 11/12, 80-233 Gdańsk, Poland; hirsch.marzena@gmail.com (M.H.); majchrowiczdaria@gmail.com (D.M.); pwierzba@eti.pg.gda.pl (P.W.); 2 Institut Européen des Membranes, UMR-5635, Université de Montpellier, École Nationale Supérieure de Chimie de Montpellier, Centre national de la recherche scientifique, Place Eugène Bataillon, Montpellier 34095, France; matthieu.weber@umontpellier.fr (M.W.); mikhael.bechelany@univ-montp2.fr (M.B.)

**Keywords:** Fabry-Pérot interferometers, atomic layer deposition, titanium dioxide thin film, fiber-optic sensor, interference

## Abstract

Fiber-optic Fabry-Pérot interferometers (FPI) can be applied as optical sensors, and excellent measurement sensitivity can be obtained by fine-tuning the interferometer design. In this work, we evaluate the ability of selected dielectric thin films to optimize the reflectivity of the Fabry-Pérot cavity. The spectral reflectance and transmittance of dielectric films made of titanium dioxide (TiO_2_) and aluminum oxide (Al_2_O_3_) with thicknesses from 30 to 220 nm have been evaluated numerically and compared. TiO_2_ films were found to be the most promising candidates for the tuning of FPI reflectivity. In order to verify and illustrate the results of modelling, TiO_2_ films with the thickness of 80 nm have been deposited on the tip of a single-mode optical fiber by atomic layer deposition (ALD). The thickness, the structure, and the chemical properties of the films have been determined. The ability of the selected TiO_2_ films to modify the reflectivity of the Fabry-Pérot cavity, to provide protection of the fibers from aggressive environments, and to create multi-cavity interferometric sensors in FPI has then been studied. The presented sensor exhibits an ability to measure refractive index in the range close to that of silica glass fiber, where sensors without reflective films do not work, as was demonstrated by the measurement of the refractive index of benzene. This opens up the prospects of applying the investigated sensor in biosensing, which we confirmed by measuring the refractive index of hemoglobin and glucose.

## 1. Introduction

Optoelectronic instruments based on spectroscopic techniques (e.g., absorption [1,2], Raman [3,4], optical tomography [5,6]) are currently applied in medicine, especially for diagnosis and imaging. However, these measurement methods are expensive, as they often require high-end measurement equipment, expensive consumables (e.g., reagents, dedicated trays, or substrates) and complex methods of sample preparation. Moreover, highly skilled laboratory staff is needed in order to ensure the quality of the measurements performed, which considerably restricts the use of optoelectronic methods in medical diagnosis.

Therefore, there is a real need to develop relatively inexpensive measuring devices that can be used by hospital staff, preferably at the point-of-care (e.g., bedside).

The use of fiber-optic sensors as low-cost measuring devices in biology and medicine represent an attractive alternative. This type of sensor has been the subject of intense research, as it offers several advantages over conventional sensors, such as very high resolution, high accuracy, and small dimensions [7,8]. In fact, fiber-optic sensors possess several advantages in comparison to electronic sensors. Their design often makes use of dielectric materials, which makes them insensitive to electric and magnetic fields generated by other medical devices. Furthermore, they are resistant to most chemical reagents and ionizing radiations. Fiber-optic sensors can be tailored to measure various species and quantities, and they are inexpensive to produce. Finally, the small dimensions of such sensors (below hundreds of micrometers) reduce their impact on the investigated area, allowing for extremely precise measurements [9,10].

Low-coherence interferometry is an excellent detection technique, as it enables the fiber-optic sensors to be insensitive to changes in the intensity of optical signal in the transmission system (because all the information about the measured values is included in the frequency component of the measuring signal spectrum [11,12]). Fiber-optic Fabry-Pérot interferometers (FPI) can be used as efficient optical sensors, and excellent measurement sensitivity can be obtained by the fine-tuning of the interferometer design [13,14,15].

In the recent years, the advancement of nanotechnology has opened new routes to manufacture thin-films, nanostructures, and nanocomposites materials. Innovative techniques which exploit these routes allow for the elaboration of precise (nano)structures to be achieved, stimulating interest in the properties of these materials and their potential applications.

Atomic layer deposition (ALD) is a vapor phase deposition technique enabling the synthesis of ultrathin films of inorganic materials, with a subnanometer thickness control [16]. ALD can be used to coat 3D substrates with a conformal and uniform film of a high-quality material, a unique capability amongst thin film deposition techniques. Consequently, ALD-grown materials can be applied in various applications such as microelectronics [17], photovoltaics [18], or optical sensing [19]. ALD is based on self-limiting reactions taking place at the surface of the substrate in a cycle-wise fashion. A typical ALD cycle consists of alternate pulses of a precursor and co-reactant gasses in the reactor chamber, separated by purge steps. The properties of the synthetized nanostructures can be tuned by adjusting the process conditions—e.g., the chemistry of the precursor(s) and the co-reactants, the temperature, the number of cycles, or the nature of the substrate [20,21,22,23].

**Figure 1:**
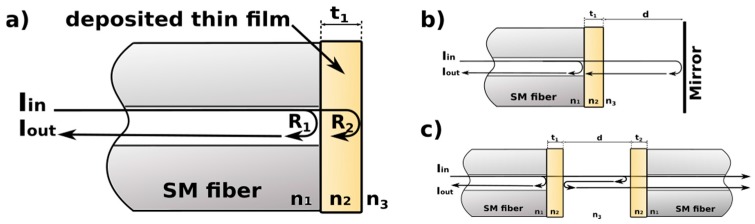
Different designs of fiber-optic Fabry-Pérot interferometers: (**a**) Interferometer with thin film sensing cavity; (**b**) and (**c**) Interferometers with extrinsic sensing cavity operating in reflective and transmission modes, respectively. *n*_1_—refractive index of the fiber; *n*_2_—refractive index of the film; *n*_3_—refractive index of the medium in the cavity; *t*_1_, *t*_2_—thickness of the thin films; *d*—length of the cavity.

Novel interferometer layouts can be considered (as depicted in Figure 1), where a thin film is grown on the end-face of a standard single-mode (SM) fiber. This film deposition creates two distinctive layouts. In the first layout, the deposited film itself is the sensing cavity of the Fabry-Pérot interferometer (Figure 1a). In the second layout, the deposited film acts as a partially transparent mirror improving the reflection at the boundary between the extrinsic cavity and the optical fiber (Figure 1b). Another view of this second layout is the fact that an extrinsic cavity is delimited by two single-mode fibers with the deposited films (Figure 1c).

In this work, we investigated the possible applications of ALD-grown films on SM fibers in low-coherence fiber-optic Fabry-Pérot sensing interferometers. We evaluate the ability of different thin films—namely titanium dioxide (TiO_2_) and aluminum oxide (Al_2_O_3_)—with different thicknesses to tune the reflectivity of the Fabry-Pérot cavity, and we illustrate this modelling work with an experimental fiber using a TiO_2_ film.

We first introduce the theory behind the concepts of FPI. Our theoretical modelling work is focused on the first interferometer layout (as shown in Figure 1a), since its properties bring a valuable insight to the expected performance of the other layout, especially the one depicted in Figure 1b. Next, as the best modelling results were obtained with TiO_2_ films, we experimentally investigated the ability of an ALD-grown TiO_2_ thin film to tune the reflectivity of the FPI cavity, by measuring the influence of TiO_2_ thin films on the intensity of reflected interfering beams. Following that, we performed measurements of the refractive index of water, benzene, hemoglobin, and glucose. The main objective of this part of the study was assessment of suitability of the sensor for applications in biosensing. Short-term stability of the TiO_2_ layers was checked and the refractive index values were compared with reference values to ascertain the correct operation of the sensor.

The investigated materials Al_2_O_3_ and TiO_2_ belong to the metal oxides group. These materials are both transparent, and they recently became the subject of growing interest for electronic and optoelectronic sensors. In fact, the sensing properties of oxide thin films are exploited as gas, humidity, and temperature sensors, as well as biosensors [24,25,26].

## 2. Theory

The Fabry-Pérot interferometers (FPIs) presented in Figure 1b,c can be considered as multi-cavity interferometers. The propagation of optical radiation in such interferometers is often described using the Gaussian beam formalism. However, FPIs with cavities formed by films manufactured by ALD can be analyzed using a simplified model. As the thickness of the deposited films is limited and as the refractive index difference between the film and the media is relatively small, the Gaussian beam propagating in such films does not expand appreciably. Consequently, in the model, the films manufactured by ALD can be replaced by planar reflective surfaces, whose reflection and transmission coefficients can be calculated using the plane wave approach [27]. This results in a tractable single-cavity model in which the information about the cavities formed by the deposited films is preserved in the values of corresponding reflection and transmission coefficients.

The reflectivity *R*_1_ at the boundary of the optical fiber and the deposited film, and the reflectivity *R*_2_ at the boundary between the film and the surrounding medium, can be calculated using the Fresnel equations:

(1) $$R_{1}=\;{\left(\frac{n_{2}-n_{1}}{n_{1}+n_{2}}\;\right)}^{2},$$

(2) $$R_{2}={\left(\frac{n_{3}-n_{2}}{n_{2}+n_{3}}\right)}^{2}$$

Using (1) and (2), the reflectivity ℜ of the deposited film can be expressed as:

(3) $$ℜ=\frac{R_{1}+R_{2}-2\sqrt{R_{1}R_{2}}\cos\delta }{1+R_{1}R_{2}-2\sqrt{R_{1}R_{2}}\cos\delta }$$

The phase difference introduced in the interferometer is expressed by Equation (4):

(4) $$\delta =\frac{4\pi }{\lambda }tn_{2}$$

where *λ*—wavelength; *t*—thickness of the deposited film; *n*_1,2,3_—refractive indices of the optical fiber core, the deposited film, and the surrounding media (as shown in Figure 1).

Establishing a precise value of the refractive index for the thin films is challenging, as it depends on the thickness and the growth conditions. Furthermore, some materials exhibit birefringence in the bulk form, whereas the thin films behave as isotropic materials [28,29,30].

Considering Al_2_O_3_ material, the values of the refractive index reported in the literature exhibit a substantial dependence on the deposition method and growth conditions [18,19,20]. In our model, the refractive index of Al_2_O_3_ was based on our group experimental data and calculated from Equation (5) [23,31]:

(5) $$n_{Al_{2}O_{3}}^{2}=1+\frac{1.4313493\lambda^{2}}{\lambda^{2}-{\left(0.0726631\right)}^{2}}+\frac{0.65054713\lambda^{2}}{\lambda^{2}-{\left(0.1193242\right)}^{2}}+\frac{5.3414021\lambda^{2}}{\lambda^{2}-{\left(18.028251\right)}^{2}}.$$

Next, the Refractive index of TiO_2_ was calculated, using Equation (6) below [32]:

(6) $$n_{TiO_{2}}^{2}=5.193+\frac{0.2441}{\lambda^{2}-0.0803}.$$

The wavelengths *λ* are expressed in µm in Equations (5) and (6).

## 3. Modelling

**Figure 2:**
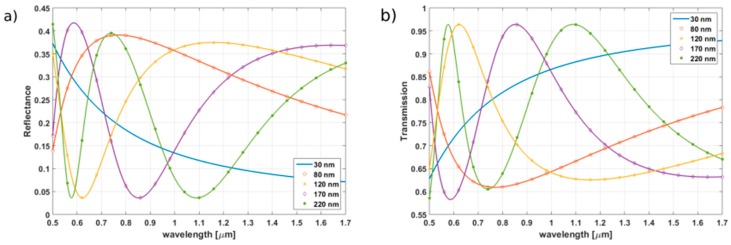
(**a**) Calculated reflectance and (**b**) calculated transmission for TiO_2_ films of thicknesses ranging from 30 to 220 nm.

**Figure 3:**
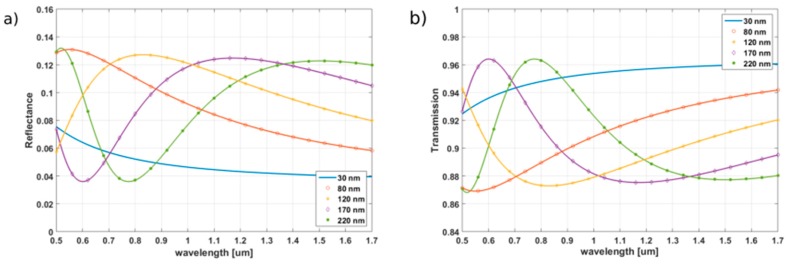
(**a**) Calculated reflectance and (**b**) calculated transmission for Al_2_O_3_ films of thicknesses ranging from 30 to 220 nm.

The spectral characteristics of the TiO_2_ and Al_2_O_3_ thin films have been calculated using the equations presented in Section 2 for various thicknesses (30, 80, 120, 170, and 220 nm). The refractive index of the medium in the cavity n_3_ was set to 1.00. The reflectance and transmission have been studied for wavelengths in the range from 500 nm to 1700 nm. The results obtained for TiO_2_ and Al_2_O_3_ films are presented in Figure 2 and Figure 3, respectively.

For the thinnest films of both oxides (30 nm), it can be seen that the spectral reflectance slowly decreases when increasing the wavelength. However, for thicker films, the optical behavior is different, as wide but distinct fringes can be noticed (for both oxides). Considering the same film thickness, the fringes of the reflectance appear faster for materials presenting higher refractive indexes (in our calculations, the assumed values of n are 2.46 for TiO_2_ and 1.75 for Al_2_O_3_ (at 1300 nm)). The fringes present in the spectra obtained for thicker films are due to the interferences taking place in the film. This effect can be exploited for the design of an interferometer where the thin film itself acts as the sensing medium. However, the measurement techniques most commonly used to process signals from FPI require that the spectrum of the source should cover at least half of the fringe [27]. This condition is difficult to fulfil for the investigation of thin films, as it would require a broadband source with a spectral width over 150 nm, which is difficult to implement. The wavelength range considered is also not very convenient for standard telecommunication optical fibers. Therefore, extracting the data from a FPI sensor using such a thin film as an active medium would require adapting the standard technologies currently used for spectral analysis. However, when considering the sensitivity of TiO_2_ thin films to humidity, temperature, and specific chemical compounds, this material could find applications in such Fabry-Pérot sensors.

The maximum reflectance obtained is higher for TiO_2_ (up to 0.417) then for Al_2_O_3_ (*R*_max_ = 0.131). However, even if the reflectance value predicted for Al_2_O_3_ thin films is lower than the one of TiO_2_, it still provides significantly higher level of reflection than a simple boundary between an optical fiber and air cavity (*R* = 0.036), especially for films thicker than 100 nm.

When considering standard single-mode optical fibers such as SMF-28, wavelengths between 1200 and 1600 nm are typically used. In this range, the maximum reflectance is achieved for films presenting thicknesses between 120 and 220 nm (Table 1).

The numerical values presented in the Table 1 were determined for the highest reflectance at selected wavelengths (the ones that are most commonly used when working with optical fibers). However, even if a much thinner film is used, the reflectance of the interferometer’s mirror will significantly increase compared to the one of a clean-cut fiber.

Considering the time-consuming nature of the ALD process, where the thickness of the layer depends on the number of performed cycles, using a thinner film allows us to considerably minimize the time required for the fabrication of the sensor. Taking this point into consideration for the experimental evaluation of the performance of ALD layer in optical fiber FPI, a thin TiO_2_ film of 80 nm thickness has been prepared on the tip of an optical fiber.

## 4. Materials

### 4.1. ALD of TiO_2_

All depositions have been carried out in a custom-built ALD reactor described elsewhere [33]. Titanium isopropoxide ((*i*PrO)_4_Ti) precursor was purchased from Sigma Aldrich and used as received. The co-reactant was millipore water. The substrates used were p-type (100) silicon wafers (MEMC Korea Company, Cheonan, South Korea) and SMF-28 optical fibers (Thorlabs, Newton, MA, USA). To remove the organic contaminants, the substrates were pre-cleaned in acetone and ethanol, and de-ionized water for 5 min in an ultrasonic bath before the depositions.

ALD of TiO_2_ was achieved using sequential exposures of (iPrO)_4_Ti and H_2_O at 120 °C separated by purge steps of argon with a flow rate of 100 sccm. The process consisted of 5 s pulse ((iPrO)_4_Ti), 30 s exposure, and 40 s purge with dry argon and a 3 s pulse (H_2_O), 30 s exposure and 60 s purge. 4000 ALD cycles were carried out in order to achieve the deposition of TiO_2_ of ≈80 nm.

Interestingly, it has been shown in previous studies that the amorphous, anatase, and rutile phases of TiO_2_ can be obtained by tuning the ALD process parameters, and that the films with different crystallinity phases presented different optical properties [34]. Furthermore, the refractive index of the film has been determined by spectroscopic ellipsometry and a value of 2.0 has been obtained (at lambda = 633 nm). This value is in agreement with the ones found in the literature for TiO_2_ films prepared with similar ALD processes (n increases typically from 2.0 to 2.5 with the increasing deposition temperature).

### 4.2. Characterization of the Films

Chemical and structural characterizations have been performed using Scanning Electron Microscopy (SEM, Hitachi S-4800, Tokyo, Japan), X-ray diffraction (PANAlytical Xpert-PRO diffractometer equipped with an X’celerator detector using Ni-filtered Cu-radiation, Almelo, The Netherlands), and Raman (Raman OMARS 89 (DILOR), Kyoto, Japan). To determine the TiO_2_ film thickness after the ALD deposition, ex-situ spectroscopic ellipsometry (SE) measurements were carried out using a Semilab GES5E visible ellipsometer (1.2–5.0 eV) at an angle of incidence of 70.1°. For all the films, the empirical Cauchy dispersion formula has been adopted to model the optical properties and the thicknesses.

### 4.3. Properties of the TiO_2_ Film

**Figure 4:**
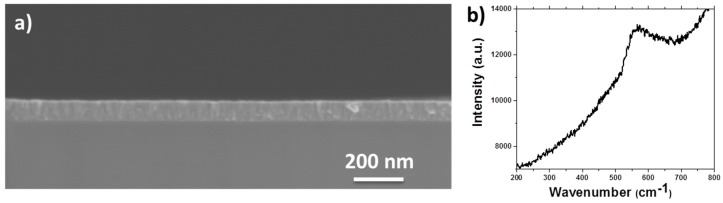
(**a**) SEM cross section image of TiO_2_ films deposited by ALD on Si substrates and (**b**) Raman spectrum of TiO_2_ films deposited by ALD.

Figure 4a shows the SEM image of a ALD-grown TiO_2_ film deposited on Si substrate after 4000 ALD cycles. The conformal coating of the Si substrate by the ALD TiO_2_ film can be clearly seen.

Spectroscopic ellipsometry (SE) measurements were carried out to evaluate the TiO_2_ film thickness as well, and for this specific sample, a thickness of 82 ± 2 nm has been obtained.

The crystallinity study that we carried out showed that the ALD films prepared were amorphous. In fact, grazing-incidence XRD measurements have been realized and the absence of peaks in the spectra obtained suggested that the as-deposited TiO_2_ film at 120 °C was amorphous. The formation the amorphous TiO_2_ phase was further confirmed by Raman spectroscopy (Figure 4b), since the Raman spectra of the TiO_2_ films deposited did not show any peaks either.

This result is in agreement with previous studies that showed that TiO_2_ films deposited by ALD below 200 °C are amorphous, and that annealing at temperatures above 300 °C are typically required to obtain the crystallization of the films. Crystalline TiO_2_ typically exhibits the anatase phase, but the rutile phase can also be achieved by ALD, using ozone or plasmas as coreactants [33,35,36]. The relative density of the ALD TiO_2_ has been reported elsewhere to be 3.6 g/cm^3^ [37].

## 5. Measurements

**Figure 5:**
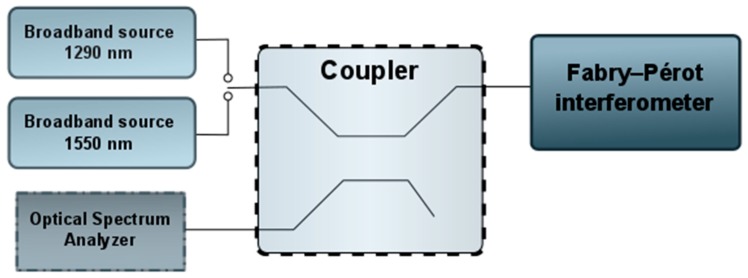
Design of the experimental sensor.

After the deposition of an 80 nm TiO_2_ film on SMF-28 optical fibers (Thorlabs, Newton, MA, USA) has been carried out, the performance of the this ALD film in a Fabry-Pérot interferometer has been tested. For this purpose, a low-coherence interferometric sensor has been used (the design of this experimental sensor is depicted in Figure 5). The sensor consists of a Fabry-Pérot interferometer working in reflective mode, an optical spectrum analyzer (Ando AQ6319, Tokyo, Japan), broadband NIR-radiation sources (S1300-G-I-20: *λ* = 1290 nm, Δ*λ*_FWHM_ = 50 nm and S-1550-G-I-20: *λ* = 1550 nm, Δλ_FWHM_ = 45 nm Superlum), and a single-mode 2 × 2 coupler with 50: 50 power splitting ratio. The standard telecommunication single-mode optical fiber (SMF-28, Thorlabs) coated by ALD is used to connect all components of the setup.

In our study, two Fabry-Pérot interferometers working in reflective mode and having a tunable cavity length were used. The first one had an 80 nm TiO_2_ thin film deposited on the fiber end face, as shown in Figure 1b. The second FPI had no film deposited and was used as the reference. The measurement was performed in two steps. First, the cavity of the FPI was set to a known length. Then, the spectrum of the light reflected from the FPI was recorded. The measurements were performed for cavity lengths ranging from 50 µm to 500 µm, yielding a series of spectra for each interferometer.

## 6. Results

In this study, the influence of TiO_2_ thin films on the quality of the spectrum reflected from the Fabry-Pérot interferometers was investigated. As the optimization function, the visibility *V* of the measured signal was used. V was defined as:

(7) $$V=\frac{I_{max}-I_{min}}{I_{max}+I_{min}}$$

where *I_max_* is the maximum intensity of the optical signal, *I_min_* the minimum intensity of the optical signal.

This choice of the optimization function is dictated by the fact that FPI used in low-coherence sensors with spectral detection should be manufactured in such a way that the peaks on the spectral characteristics recorded by the detection setup have the maximum amplitude, i.e., maximum visibility V defined by (7) (in an ideal case V should be equal to 1.0). This contributes to the maximum accuracy of data processing algorithms in these sensors. When V decreases, the accuracy of these algorithms may degrade, although not significantly, as long as V is above 0.7–0.8. Values of *V* corresponding to optimal cavity length *l_opt_* and to 50% and 200% of *l_opt_* are shown in Table 2, for each interferometer and each wavelength.

It is important to note that the optimal length *l_opt_* of the cavity of the Fabry-Pérot interferometer with TiO_2_ thin film is around 100 μm and for the Fabry-Pérot interferometer with no film is around 200 μm. The corresponding visibility values are at least 0.95 for both light sources.

**Figure 6:**
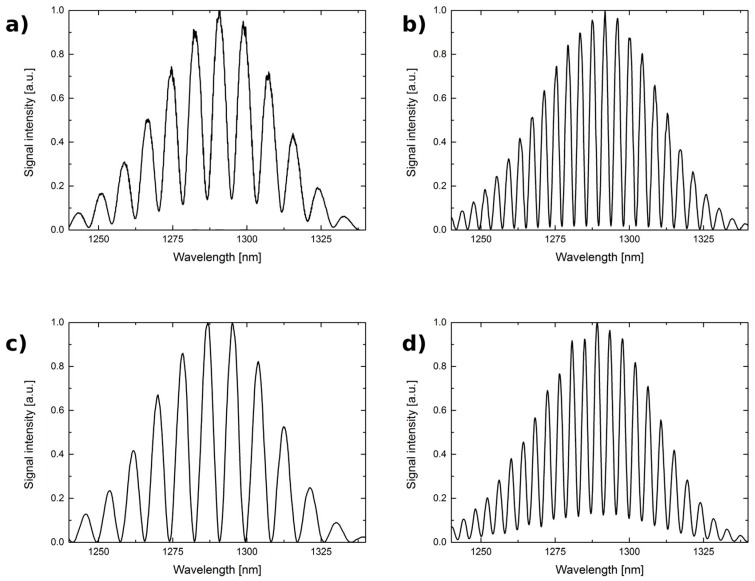
The measurement signal for the 1290 nm source. The Fabry-Pérot interferometer made by: optical fiber and cavity length: (**a**) 100 μm; (**b**) 200 μm; optical fiber with TiO_2_ thin film and cavity length: (**c**) 100 μm; (**d**) 200 μm.

**Figure 7:**
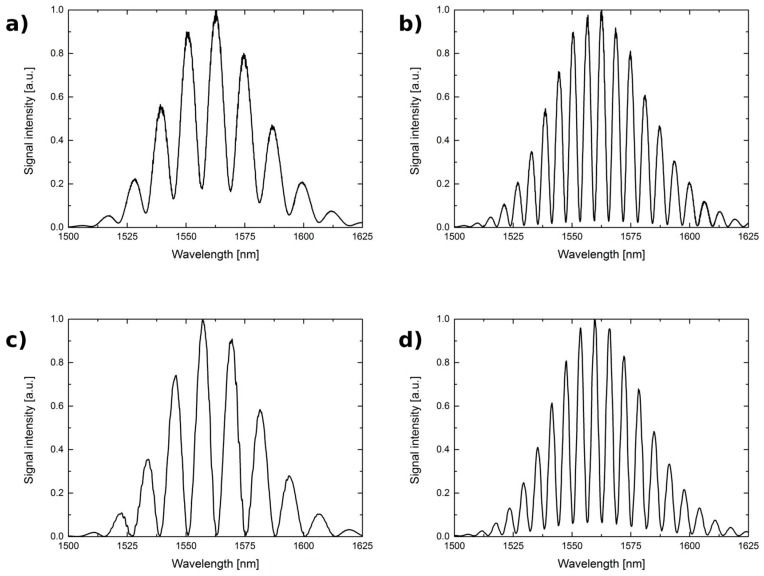
The measurement signal for the 1550 nm source. The Fabry-Pérot interferometer made by: optical fiber and cavity length: (**a**) 100 μm; (**b**) 200 μm; optical fiber with TiO_2_ thin film and cavity length: (**c**) 100 μm; (**d**) 200 μm.

It can be seen that the visibility does not fall steeply. In particular, the visibility for 0.5 *l_opt_* and 2.0 *l_opt_* does not decrease below 0.7 for any investigated Fabry-Pérot interferometer. This indicates that the cavity length of such an interferometer can be varied in a relatively broad range, up to 4:1, without significant degradation of the performance of the sensor. The measured spectra obtained with the cavity length equal to 100 µm and 200 µm are presented in Figure 6 and Figure 7.

In order to confirm that proposed ALD-enhanced FPI can be used for measurements of the materials with refractive index close to that of silica glass, sensor response was examined for measurements of benzene (refractive index equal 1.4769 at 1550 nm, while for the core of SMF-28 optical fiber it is 1.468). The calculated value of refractive index obtained from recorded spectra and the reference data are shown to be in good agreement, with difference below 0.029. Following, the measurements were performed for pure water. The value of refractive index measured by our sensor was within 0.032 of the reference value.

**Figure 8:**
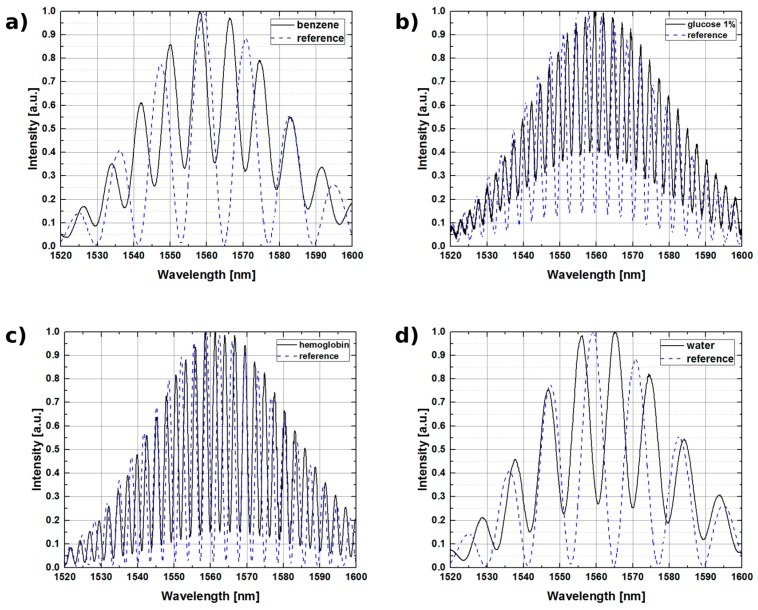
Measurements of Fabry-Pérot interferometer carried out with an optical fiber coated with TiO_2_ layer when the cavity is filled with: (**a**) benzene; (**b**) glucose (1% solution); (**c**) hemoglobin (13.4 g/dL); and (**d**) water.

To further explore the potential of presented construction in the biosensing applications, the fabricated sensor head was tested with sample of glucose and hemoglobin solution, obtaining refractive index values of 1.3940 and 1.2958, respectively. All of the measurements were performed with the 1550 nm source, the obtained spectra are presented in Figure 8.

The visibility V of the measured signals has decreased in all cases, which was expected as the cavity length was optimized for mediums with a refractive index of 1.0. However, this reduced visibility should not degrade the accuracy of the refractive index calculations.

For each sample, the measurements were performed during a 7-h period. The spectra recorded for each liquid remained stable during this time period. This initial stability test indicated that there was no degradation in the sensing properties of the TiO_2_ film.

## 7. Conclusions

In the present study, the application of oxide ultrathin films in low coherence fiber-optic Fabry-Pérot sensing interferometers was investigated. The thin films on the tip of SM fibers were aimed to tune the reflectivity of the FPI cavity. The reflectance and transmission spectra were modelled for TiO_2_ and Al_2_O_3_ films of various thicknesses. The obtained results indicate that it is possible to use thin TiO_2_ film of a thickness around 200–300 nm as active medium in a Fabry-Pérot interferometer. However, the measurements require either an extremely broadband source or a specific adapted signal processing technique. TiO_2_ and Al_2_O_3_ thin films of 100–200 nm deposited on the tip of the optical fiber can also be used as semi-reflective surfaces in order to improve the performance of extrinsic Fabry-Pérot interferometers.

Experiments with a Fabry-Pérot interferometer working in reflective mode were performed in order to illustrate and to verify the modelling presented. The cavity of the interferometer was delimited by an 80 nm TiO_2_ film deposited by ALD on the fiber end face and by a silver mirror. The cavity length of the interferometer corresponding to the maximum fringe visibility was 100 μm. The reference interferometer (without the TiO_2_ film) presented an optimal length of 200 μm. Moreover, the level of the signal reflected from the coated interferometer was two times higher than the one from the reference interferometer.

The measurement of refractive index of benzene, which is close to that of silica glass fiber, did not result in any appreciable signal deterioration and yielded a result within 0.029 from the reference value. This confirms the ability of the presented sensor to operate in the refractive index range close to that of silica glass, where sensors without reflective films do not work. Based on the measurement results of refractive index of air and water it can be concluded that the measurement range of our sensor extends from 1.0 to at least 1.5, which gives the sensor good application prospects in biosensing. These prospects were further enhanced by measuring the refractive index of hemoglobin and glucose, during which no degradation in the sensing properties of the TiO_2_ film was observed.

## Figures and Tables

**Table 1:** Comparison of calculated values of reflectance and optimal film thickness *t* chosen for the highest reflection at selected wavelengths.

*λ* (nm)	*R*_TiO_2__	*R*_Al_2_O_3__
900	0.3769, *t* = 80 nm	0.1258, *t* = 120 nm
1300	0.3679, *t* = 120 nm	0.1226, *t* = 170 nm
1550	0.3663, *t* = 220 nm	0.1225, *t* = 220 nm

**Table 2:** Visibility of the measured signal in the Fabry-Pérot interferometer.

Fabry-Pérot Interferometer Made by	Length of the Fabry-Pérot Cavity	Central Wavelength of Light Source 1290 nm	Central Wavelength of Light Source 1550 nm
Optical fiber	100 μm	0.75	0.70
	200 μm	0.96	0.95
	400 μm	0.77	0.87
Optical fiber with TiO_2_ thin film	50 μm	0.89	0.84
	100 μm	0.99	0.98
	200 μm	0.8	0.88
